# Sleep/Wake Disruption in a Mouse Model of BLOC-1 Deficiency

**DOI:** 10.3389/fnins.2018.00759

**Published:** 2018-11-15

**Authors:** Frank Y. Lee, Huei-Bin Wang, Olivia N. Hitchcock, Dawn Hsiao Loh, Daniel S. Whittaker, Yoon-Sik Kim, Achilles Aiken, Collette Kokikian, Esteban C. Dell’Angelica, Christopher S. Colwell, Cristina A. Ghiani

**Affiliations:** ^1^Department of Human Genetics, David Geffen School of Medicine, University of California, Los Angeles, Los Angeles, CA, United States; ^2^Molecular, Cellular, & Integrative Physiology Program, University of California, Los Angeles, Los Angeles, CA, United States; ^3^Integrative Biology and Physiology Program, University of California, Los Angeles, Los Angeles, CA, United States; ^4^Department of Psychiatry and Biobehavioral Sciences, David Geffen School of Medicine, University of California, Los Angeles, Los Angeles, CA, United States; ^5^Department of Pathology and Laboratory Medicine, David Geffen School of Medicine, University of California, Los Angeles, Los Angeles, CA, United States

**Keywords:** BLOC-1, sleep, circadian rhythms, dysbindin, pallidin, CREB, intellectual developmental disabilities

## Abstract

Mice lacking a functional Biogenesis of Lysosome-related Organelles Complex 1 (BLOC-1), such as those of the pallid line, display cognitive and behavioural impairments reminiscent of those presented by individuals with intellectual and developmental disabilities. Although disturbances in the sleep/wake cycle are commonly lamented by these individuals, the underlying mechanisms, including the possible role of the circadian timing system, are still unknown. In this paper, we have explored sleep/circadian malfunctions and underlying mechanisms in BLOC-1-deficient pallid mice. These mutants exhibited less sleep behaviour in the beginning of the resting phase than wild-type mice with a more broken sleeping pattern in normal light-dark conditions. Furthermore, the strength of the activity rhythms in the mutants were reduced with significantly more fragmentation and lower precision than in age-matched controls. These symptoms were accompanied by an abnormal preference for the open arm in the elevated plus maze in the day and poor performance in the novel object recognition at night. At the level of the central circadian clock (the suprachiasmatic nucleus, SCN), loss of BLOC-1 caused subtle morphological changes including a larger SCN and increased expression of the relative levels of the clock gene *Per2* product during the day but did not affect the neuronal activity rhythms. In the hippocampus, the pallid mice presented with anomalies in the cytoarchitecture of the Dentate Gyrus granule cells, but not in CA1 pyramidal neurones, along with altered PER2 protein levels as well as reduced pCREB/tCREB ratio during the day. Our findings suggest that lack of BLOC-1 in mice disrupts the sleep/wake cycle and performance in behavioural tests associated with specific alterations in cytoarchitecture and protein expression.

## Introduction

BLOC-1 (Biogenesis of Lysosome-related Organelles Complex-1) is a ubiquitously expressed, stable octameric protein complex containing, among other subunits, dysbindin and pallidin (reviewed by Ghiani and Dell’Angelica, 2011; Mullin et al., 2011). Advances have been made in unravelling the function of this protein complex in the biogenesis of lysosome-related organelles and, at least, of a subset of recycling endosomes (Di Pietro et al., 2006; Setty et al., 2007; Newell-Litwa et al., 2010; Delevoye et al., 2016; reviewed by Dell’Angelica, 2016; Hartwig et al., 2018). Specifically, BLOC-1 is required for efficient formation of membrane tubules from vacuolar sorting endosomes through coordination of the tubule-forming activity of kinesin family member 13A and the tubule-detachment activity of annexin A2 and the actin cytoskeleton (Delevoye et al., 2016); these tubular structures bear the characteristics of recycling endosomes and, depending on cell type, may deliver integral membrane proteins to intracellular compartments such as melanosomes (Di Pietro et al., 2006; Setty et al., 2007; Sitaram et al., 2012) and synaptic vesicles (Newell-Litwa et al., 2009) as well as to primary cilia (Monis et al., 2017) and neurites (Larimore et al., 2011). While it has been proposed that alternatively spliced isoforms of the dysbindin subunit might fullfil cellular functions independently of BLOC-1 (Wang et al., 2014), the protein pallidin is thought to function exclusively as a component of BLOC-1 (reviewed by Ghiani and Dell’Angelica, 2011). In humans, mutations in the genes encoding three BLOC-1 subunits, including dysbindin and pallidin, lead to a rare autosomal recessive disorder named Hermansky-Pudlak syndrome (HPS), which is characterised by deficient function of specialised lysosome-related organelles such as melanosomes and platelet dense granules with consequent presentations of albinism and prolonged bleeding times (reviewed by Wei, 2006; Huizing et al., 2008; Wei and Li, 2013; El-Chemaly and Young, 2016). Mouse models of BLOC-1-deficiency recapitulate the pathophysiological traits of HPS, but also display behavioural phenotypes suggesting a possible involvement of this complex in brain development and function(s). Although controversial, genetic variations in BLOC-1 subunits have been linked to alterations in cognitive abilities and increased susceptibility for neurodevelopmental psychiatric disorders in humans (Kircher et al., 2009; Luciano et al., 2009; Narr et al., 2009; Varela-Gomez et al., 2015; reviewed by Hartwig et al., 2018). BLOC-1 expression in the mouse central nervous system is developmentally regulated with higher expression during perinatal periods (Ghiani et al., 2010), and in its absence, hippocampal neurones in cultures exhibit defective neurite outgrowth (Ghiani et al., 2010; Ito et al., 2010; Ma et al., 2011). Behavioural and neurophysiological abnormalities have been reported in the sandy mouse, a naturally occuring mutant carrying an in-frame deletion in the gene encoding dysbindin (reviewed by Ghiani and Dell’Angelica, 2011, for examples see Bhardwaj et al., 2009; Cox et al., 2009; Ryder and Faundez, 2009; Talbot, 2009; Papaleo et al., 2012; Carr et al., 2013; Glen et al., 2014; Larimore et al., 2014, 2017; Bhardwaj et al., 2015a; Yuan et al., 2016) as well as in other dysbindin-mutants (Petit et al., 2017; Chang et al., 2018). Importantly, Spiegel et al. (2015) reported that the pallid and sandy lines show similar impairment of social recognition memory. Consistently, *Drosophila* *melanogaster* lacking functional BLOC-1 display impaired neurotransmission and altered behaviour (Cheli et al., 2010; Shao et al., 2011; Dickman et al., 2012; Mullin et al., 2015; Chen et al., 2017). These findings support the proposed argument that mutations affecting BLOC-1 stability elicit cognitive and behavioural deficits. Recently, a 6-year-old male has been identified as BLOC-1-deficient (due to a mutation in the dysbindin-encoding gene) and presenting with the symptoms of HPS as well as with motor and language developmental delays (Bryan et al., 2017), and a 52-year-old female has been described as deficient in the same complex (due to a mutation in the pallidin-encoding gene) and presenting with HPS together with schizophrenia (Okamura et al., 2018).

Individuals with neurodevelopmental psychiatric disorders often exhibit a dysregulated sleep/wake cycle (reviewed by Robinson-Shelton and Malow, 2016; for examples see Couturier et al., 2005; Johnson et al., 2009; Sivertsen et al., 2012), which may be driven by a malfunctioning circadian system. The molecular clockwork that drives circadian oscillations is not only expressed in the central circadian clock, the suprachiasmatic nucleus (SCN), but also in other brain areas, including some highly relevant to intellectual and developmental disabilities (IDD). A variety of studies has suggested that disturbed sleep exacerbates IDD-related symptoms such as impaired social interactions, presence of repetitive behaviours, mood disorders, and inattention or hyperactivity (reviewed by Schreck et al., 2004; Gabriels et al., 2005; Goldman et al., 2009). Although symptoms of dysregulated sleep/wake cycle are common and robust, the underlying mechanisms including the possible role of a faulty central clock are difficult to assess in IDD patients. Prior work found evidence for a disrupted sleep/wake cycle in the sandy mouse, although only under abnormal conditions of constant light (Bhardwaj et al., 2015b). Mutations in BLOC-1 subunits were reported to cause broadly similar phenotypes; however, important differences between the mutant lines were also observed (Larimore et al., 2014; Spiegel et al., 2015). In the present study, we explored behavioural sleep and locomotor activity rhythms in adult BLOC-1-deficient, pallid mice, as well as the presence of pathophysiological alterations or disorganisation in the SCN. The circadian clock modulates cognition and drives rhythms in signalling pathway(s) in IDD-related brain areas, such as the hippocampus (Stephan and Kovacevic, 1978; Wang et al., 2009; Phan et al., 2011; Fernandez et al., 2014; Shimizu et al., 2016). Hence, we determined whether the rhythmic regulation of clock protein expressions and signalling were altered in the pallid hippocampus.

## Materials and Methods

### Animals

All experimental protocols used in this study were approved by the University of California, Los Angeles (UCLA) Animal Research Committee. UCLA Division of Laboratory animal recommendations for animal use and welfare, as well as National Institutes of Health guidelines, were followed. BLOC-1-deficient male pallid (B6.Cg-*Bloc1s6^pa^*/J)^1^ and “wild-type” (WT) control strain (C57BL/6J)^2^ were from our breeding colony maintained at UCLA. The pallid strain carries a non-sense mutation in the *Blos1s6* gene (also known as *Pldn*) encoding pallidin (Huang et al., 1999), which is an essential component of BLOC-1 (Falcón-Pérez et al., 2002; Moriyama and Bonifacino, 2002), while carrying no mutations in the *Dtnbp1* gene encoding dysbindin.

### Behavioural Tests

#### Video-Recorded Sleep Behaviour

Behaviour was measured with video recording in combination with an automated mouse tracking analysis software system as previously described (Li et al., 2015; Loh et al., 2015). WT and pallid mice (*n* = 8 animals/genotype), all males 3–5 months old (mo), were singly housed in transparent cages under a 12:12 h light-dark (LD) cycle. Mice were housed in see through plastic cages containing bedding, but without the addition of nesting material. Video capture of a side-on view of each cage was obtained, and was not obstructed by the top mounted food bin or water bottle. Cages were under constant infrared LED lighting. Video was captured using infrared surveillance cameras (700TVL SONY Effio-E with 2.8–12 mm zoom; Gadspot Inc., City of Industry, CA, United States) equipped with IR850 infrared philtre (Neewer Technology Ltd., Guangdong, China). All animals were tracked by the ANY-maze software (Stoelting Co., Wood Dale, IL, United States) and the behavioural sleep was determined when 95% of the animal remained immobile for more than 40 s. Continuous recording was performed over 4 days and data collected from days 2 and 3 were used for further analysis. Collected sleep data were exported in 1 min bins, and total amount of sleep was determined by summing the duration of sleep in the day (Zeitgeber Time, ZT0-12) or night (ZT12-24). ZT0 was the onset of lights turning on, and ZT12 was the time when lights turn off under the LD conditions. The number of sleep bouts in the day or night was counted by using ClockLab programme (Actimetrics, Wilmette, IL, United States). The sleep onset was automatically detected by the ClockLab software analysis function. Briefly, ClockLab first bins the activity or sleep record into activity/sleep bouts. One activity/sleep bout was counted when activity was separated by a gap of 21 min or more. The time of the first activity/sleep bout after at a least 6-h period of inactivity is considered the onset.

#### Cage Activity Rhythms

Methods used were similar to those described previously (Li et al., 2015; Loh et al., 2015). WT and pallid mice (males, 3–5 mo, *n* = 8–9 animals/genotype) were singly housed in cages with wheels (11.5 cm diameter, Mini Mitter, Bend, OR, United States) to record their locomotor activity, with a 12:12 h LD cycle and entrained for 2 weeks before data collection. Cage activity was then recorded for at least 10 days in LD, followed by another 14 days of constant darkness (DD) to obtain free-running activity. Wheel revolutions were recorded in 3 min bins, and 10 days of data under each condition were averaged and analysed to determine the period and rhythmic strength as previously described (Li et al., 2015; Loh et al., 2015). The locomotor activity rhythms were analysed using the periodogram analysis combined with the χ2 test with *P* = 0.05 significance level (El Temps, Barcelona, Spain)^3^ on the raw data. The periodogram is commonly used to identify the dominant periods (or frequencies) of a time series for circadian studies. It shows the amplitude (referred to as “power” or % variation) of periodicities in the time series for all periods of interest (between 20 and 31 h in 3 min steps). The power values were normalised to the percentage of variance derived from the Qp values of the periodogram (Qp × 100/N; N = total number of data points) according to the calculated *P* = 0.05 significance level. Activity amount over 24 h was determined by averaging 10 days of wheel revolutions (rev/h). Nocturnality was defined as the % of total activity within a 24-h cycle that occurred in the night. Precision was determined by calculating the daily variation in onset from a best-fit regression line drawn through 10 days of activity in both LD and DD conditions using the ClockLab programme (Actimetrics, Wilmette, IL, United States). Fragmentation was defined by the number of activity bouts per day. One activity bout was counted when activity was separated by a gap of 21 min or more. Some mice in DD were exposed to a brief light treatment (white light, 100 lux at the cage level, 10 min) at circadian time (CT) 16, where CT12 was defined by the locomotor activity onset. After light exposure, the animals were allowed to free-run undisturbed in DD for 10 days. Phase shifts in the activity rhythm were determined by measuring the phase difference between eye-fitted lines connecting the onset of activity for 10 days before and 10 days after an experimental manipulation.

#### Elevated Plus Maze Test

The elevated plus maze (EPM) test was used to assess anxiety-like behaviour and followed the protocol described by Komada et al. (2008). The maze has contralateral arms with opaque walls (closed), contralateral arms with no walls (open), and a square centre area where the arms meet. It is elevated on a tripod and cleaned with 70% alcohol between each test. Mice were tested during their resting phase between ZT2-10 under white LED lights. Light in walled arms measures 25% lower intensity than in open arms. WT mice were tested with a cream background and pallid mice with a black background to see them clearly. Tests were run for 10 min, recorded and scored using ANY-maze video-tracking software (Stoelting Co., United States). All mice were single caged and no habituation to the maze was performed. Prior to the testing, mice were acclimated to the testing room. At the start of each test, a well-handled mouse was placed at the junction of the open and closed arms, facing the open arm opposite the experimenter. Tests were started using a remote activation device, while the experimenter moved to a space partitioned from the testing area. ANY-maze automatically records distance, speed, the number of entries, and the time spent in the open, closed, and centre areas. Mice were considered to have entered any area when 85% of the body was in the area, and considered to have exited when 75% was out of the area. Data are shown as the mean ± SEM of 9–10 animals/genotype.

#### Open Field Test

The open field (OF) test was used to assess anxiety-like behaviour (Bailey and Crawley, 2009) as well as exploratory behaviour and spontaneous locomotor activity during the animals’ active phase (ZT14-16) under dim red light. Animals were individually placed in a plastic arena with opaque walls (47 cm wide × 40 cm long × 30 cm tall). The experimenter moved out of the testing area after placement of animals. OF activity was recorded for 10 min by a ceiling-mounted infrared equipped 800TVL dome video camera (101 AudioVideo Inc., Sunnyvale, CA, United States). The tests were performed under dim red light (3-5 lux). ANY-maze software was used to code the videos. Anxiety-like behaviour was scored for time spent in the centre versus the peripheral zone of the testing arena. The central 25% of the arena was designated as the centre zone. To assess exploratory behaviour and spontaneous locomotor activity, speed and total distance travelled over 10 min were scored using ANY-maze software. Data are shown as the mean ± SEM of 15–16 animals/genotype.

#### Novel Object Recognition Test

The novel object recognition (NOR) test was performed as a hippocampal-dependent cognitive test (Cohen et al., 2013). NOR tests were performed during their active phase (ZT14-16) under dim red light. The animals were individually placed in a large arena (60 × 48 × 30 cm). On day 1 and day 2, they were allowed to habituate to the empty arena for 10 min. A pair of identical objects was introduced on day 3 and day 4, the mice were allowed to explore the arena for 10 min and the time spent exploring the objects on day 3 recorded to assess their interest in the objects. On day 5, the mice were tested by replacing one familiar object with a novel object of different shape and material (plastic or glass). They were given 5 min to explore the arena and objects. Distance travelled and time spent on each object were tracked by ANY-maze software. No criterion for the minimum amount of time for object exploration was set. The animals’ performance (recognition score) was determined by the discrimination index (DI: time spent with the novel object/time spent with the familiar object + time spent with the novel object). A DI of 0.5 means the testing animals spent equal amount of time with both objects, while a higher value suggests they favour the novel object over the other familiar one. Data are shown as the mean ± SEM of 15–16 animals/genotype.

#### Resident-Intruder Test

The resident-intruder test was performed as described (Koolhaas et al., 2013) to assess the aggression in WT (*n* = 6) and pallid male mice (*n* = 7) under regular LD condition. The ‘resident’ mice were acclimated in their home cage for five min before the tests began. Unfamiliar WT mice with lighter body weights were selected as the ‘intruder’ mice. The test started when the intruder mouse was introduced to the resident’s cage and proceeded for five min. The tests were video recorded during the early night (ZT14-16) under dim red light (3–5 lux) and manually scored post-hoc for aggressive behaviours by two independent investigators. Exploration time before first attack, number of attacks, total time of attacks and the duration of the longest attack were recorded. In those cases in which no abnormal behaviour was observed, no further analysis was carried out. The scores from the two observers were averaged per mouse.

### Histoanatomical and Morphometrical Analyses of the SCN and the Hippocampus

Wild-type and pallid mice (males, 3–5 mo) were anesthetised with isoflurane at a specific time during the day or the night (ZT6 or 8 and ZT14, respectively) and transcardially perfused with phosphate-buffered saline (pH 7.4) containing 4% (w/v) paraformaldehyde (Electron Microscopy Sciences, Hatfield, PA, United States). The brains were rapidly dissected out, post-fixed overnight in 4% paraformaldehyde at 4°C, and cryoprotected in 15% sucrose. Coronal sections (30 or 50 μm) were obtained using a cryostat or a vibratome (Leica, Buffalo Grove, IL, United States), collected sequentially and then processed for Nissl staining using cresyl violet (Sigma) or immunofluorescence. The histomorphological and cytoarchitectural assessments were performed by two independent observers “masked” to genotype of the animals.

#### Nissl Staining

Coronal brain sections (30–50 μm) containing the entire left and right SCN were stained with 1% cresyl violet solution (Sigma; Li et al., 2015). Photographs were acquired on a Zeiss Axioskop equipped with a colour Axiocam using a 10x objective to include both left and right SCN, and the AxioVision software (Zeiss, Pleasanton, CA, United States). Images were used to estimate the area, height and width of both left and right SCN. Measurements (in μm) were obtained using the Zeiss software. Because the borders of the Nissl-defined SCN are somewhat arbitrary, measurements of the SCN were taken by two observers masked to the genotype and gender of the animals. For each animal, the three measurements were performed in consecutive slices of the SCN. Those from the 2 most central sections (largest area), the 2 sections anterior and 3 posterior to these were summed (a total of 7 consecutive sections). Since no significant differences between the measurements in the left and right SCN were found, these values were averaged. Data are shown as the mean ± SEM of 4 animals/genotype (ZT8).

#### Arginine Vasopressin (AVP) and Vasoactive Intestinal Peptide (VIP) Immunostaining

Free-floating coronal sections (50 μm) containing the entire SCN were blocked for 1 h at room temperature (RT) in carrier solution (1% BSA and 0.3% Triton X-100) containing 10% normal donkey serum and then incubated for 2 h at 37°C with the primary antibodies (please see Supplementary Table 1 for primary antibodies list and detailed information) diluted in carrier solution containing 5% normal donkey serum, followed by the appropriate secondary antibody conjugated to Cy3 or AlexaFluor 488 (Jackson ImmunoResearch Laboratories, Bar Harbor, ME, United States). Sections were mounted and coverslips applied onto a drop of Vectashield mounting medium containing DAPI (4′-6-diamidino-2-phenylinodole; Vector Laboratories, Burlingame, CA, United States). Analyses: Two methods of analysis were carried out on these sections. First, stereological analysis was performed by a single experimenter using a Zeiss AxioImager M2 microscope (Zeiss, Pleasanton, CA, United States) equipped with a motorised stage controlled by the StereoInvestigator software (MicroBrightField Biosciences, Williston, VT, United States). The area of interest was defined as the entire SCN, and outlined at 10x magnification using anatomical markers and cell density. Due to the SCN’s small area and the low number of VIP^+^ and AVP^+^ neurones in the SCN, stereological parameters were designed to cover the entire area of interest. All immunopositive cell bodies were counted directly using a 40x objective in serial slices containing the entire SCN (rostral to caudal). Data are shown as the mean ± SEM, *n* = 5–6 animals/genotype. Second, the relative intensity of AVP or VIP was quantified by scanning densitometry using the NIH Image Software (ImageJ) in individual positive cells in both the left and right middle SCN (largest part). Images were acquired on a Zeiss AxioImager M2 microscope equipped with an AxioCam MRm and the ApoTome imaging system, using a 10x objective and the Zeiss Zen digital imaging software. Positive cells were outlined using the oval selection tool and the area of the cell outline was kept constant/image. Data are shown as the mean ± SEM of AVP^+^ (35–69 total cells/slice) and VIP^+^ (14–38 total cells/slice) cells in two slices/animal/genotype (*n* = 5–6 animals/genotype).

#### PERIOD2 Immunostaining

Free-floating coronal sections (50 μm) containing the middle-central part of the SCN or the hippocampus were obtained using a vibratome and blocked for 1 h at RT in carrier solution (1% BSA and 0.3% Triton X-100) containing 10% normal donkey serum and then incubated overnight at RT with an antibody against PER2 (PERIOD2; Supplementary Table 1) diluted in carrier solution containing 5% normal donkey serum, followed by the secondary antibody conjugated to Cy3. Sections were mounted and coverslips applied onto a drop of Vectashield mounting medium containing DAPI. Sections were visualised on a Zeiss AxioImager M2 equipped with an AxioCam MRm and the ApoTome imaging system (Zeiss, Thornwood, NY, United States) and images that included both left and right SCN acquired using a 20x objective and the tile feature of the Zeiss Zen software. Analyses: Three methods of analysis were carried out on these sections. First, the distribution of the staining intensity was obtained for each left and right SCN separately in two consecutive slices/animal using the Profile Plot Analysis feature of ImageJ^4^. Briefly, a rectangular box of fixed size [358 μm × 617 μm (width × height)] to include the entire nucleus was set for each side of the SCN, and a column plot profile was generated whereby the *x*-axis represents the horizontal distance through the SCN (medial to lateral) and the *y*-axis represents the average pixel intensity per vertical line within the rectangular box. Subsequent processing of the resulting profiles was performed for left and right SCN images separately. To average the profiles of the two slices/animal and obtain a single curve/animal, fourth-order polynomial curves were fit to best estimate the position of the intensity peak on the *x*-axis, and using this position the original *y*-axis values were aligned and averaged arithmetically [1 profile per section (left or right), 2 sections per animal]. Data are shown as the average profile/genotype at either day or night, *n* = 3–9 animals per genotype/ZT. Second, the relative intensity of PER2 staining in the SCN was quantified in 2 slices containing both the left and right central SCN, by scanning densitometry using the AxioVision software. The largest SCN was identified and the left and right nucleus outlined. The resulting shapes were saved as region of interest and used to measure the relative fluorescence intensity in all the images. The values from the left and right SCN were averaged/slice and 2 consecutive slices per animal were used and averaged to obtain one value/animal. Data are shown as the means ± SEM of 3–9 animals per genotype/ZT. Third, the number of PER2 positive cells in the left and right SCN was counted using the “Analyse Particles” feature of ImageJ. Images were first converted to binary using the default intensity threshold. Cells were included based on an area of 25 μm^2^ or higher with no restriction on circularity. The counts from the left and right SCN from 2 consecutive slices were averaged and data are shown as the mean ± SEM of 3–9 animals per genotype/ZT.

#### Golgi Staining

Whole brains were rapidly dissected from P60 WT and pallid male mice (*n* = 6 animals/genotype) during the day (ZT6) and then processed following the FD Rapid GolgiStain Kit manufacturer’s instructions (FD NeuroTechnologies; Columbia, MD, United States). Pyramidal neurones from the Cornus Ammonis (CA1) and granule cells from the Dentate Gyrus (DG) of the hippocampus were selected based on their dark impregnation, projected and traced using a 40x objective on a Leica upright microscope (Leica Microsystems, Buffalo Grove, IL, United States) equipped with a camera lucida (Leica) (*n* = 12–15 neurones per animal/region/genotype, *n* = 6 animals/genotype). Quantitative description of the complexity of the proximal dendritic arbour of granule cell and pyramidal neurones (both apical and basal) was performed using the Sholl analysis (Sholl, 1953), using 25 μm-interval concentric circles and by placing the soma of the cells in the centre as reference. The number of crosses per shell was plotted against the shell distance from the soma. In addition, the length and number of the primary, secondary and tertiary dendrites of each neuron, as well the number of branching points/order were determined using the Axiovision software (Zeiss, version 4.8; Ghiani et al., 2010). Analyses were performed by two independent investigators masked to the genotype of the animals.

### Brain Slice Preparation and Electrophysiology

Brain slices were prepared at ZT00:00 h or ZT11:00 h. Mice (males, 3 mo) were anesthetised with isoflurane (USP, Primal healthcare) and decapitated and brains were quickly dissected out and immersed in 95% CO_2_/5% O_2_ mixed gas (mixture gas) saturated cold artificial cerebrospinal fluid (ACSF), containing (in mM): NaCl, 124; NaH_2_PO_4_, 1.25; KCl, 3; NaHCO_3_, 26; MgSO_4_, 1.3; CaCl_2_, 2.4; glucose, 10. Coronal slices (∼450 μm) containing the SCN were prepared using a vibroslicer (Leica VT1200, Leica Biosystems Inc.) and incubated at RT (23–26°C, 30–40 min) in the mixture gas-aerated ACSF. Slices were transferred to the recording chamber and continuously perfused with mixture gas-saturated warm (30°C) ACSF. Extracellular single-unit recordings were acquired between ZT1:00 h ∼ ZT12:00 h or ZT12:00 h ∼ ZT23:00 h. Borosilicate Micropipettes (<10 MΩ, WPI), filled with ACSF, were used as recording pipettes. The single-unit recordings were carried out in the whole SCN slice. At least 6 units were usually sampled every hour for a 1-min period. The voltage signals were fed serially into an Axopatch 200B (Molecular Devices, San Jose, CA, United States), and amplified (Output Gain: x 500). The collected and processed signals were digitised and sampled at 10,000 Hz intervals (Digidata1320A, pCLAMP 10.3; Molecular Devices).

To detect the time-of-peak of circadian firing activity rhythm of SCN neurons, which is a reliable marker of the phase of circadian pacemaker, the mean firing rates of single units sampled for sequential 2-h periods with 1-h lags were plotted against ZT (Kim et al., 2015). In order to quantify the difference of circadian firing rhythm, the time-of-peak detected in pallid slice was compared with the average time-of-peak of WT mice.

### Western Blot

Western blots were performed as previously described (Loh et al., 2015). Briefly, hippocampi were rapidly dissected from WT and pallid mice (males, 3–5 mo) at specific times during the day or the night (ZT2, ZT8, and ZT14) and homogenised in lysis buffer [50 mM Tris-HCl, 0.25% (w/v) sodium deoxycholate, 150 mM NaCl, 1 mM EDTA, 1% Nonidet P40, 1 mM sodium vanadate, 1 mM AEBSF, 10 μg/ml Aprotinin, 10 μg/ml Leupeptin, 10 μg/ml Pepstatin, and 1 mM sodium fluoride]. Total protein concentration in cleared extracts was estimated with the Pierce’s bicinchoninic acid Protein Assay Kit (Thermo Fisher Scientific, Carlsbad, CA, United States) using increasing concentrations of BSA as standards. Hippocampal tissue lysates from pairs of WT and mutants were generated at the same time and run in the same gel. Equal protein loading was further verified by Ponceau S solution (Sigma) reversible staining of the blots, and each extract was also analysed for relative protein levels of β-actin (Sigma). Samples were analysed for relative protein levels of PER2, total CREB (tCREB) and the phosphorylated form at Ser133 of CREB (pCREB; Supplementary Table 1). Relative levels of pCREB and tCREB were determined by stripping and reprobing the same membrane. Relative intensities of the protein bands were quantified by scanning densitometry using the NIH Image Software (ImageJ), and each value background-corrected and normalised to β-actin. Data are reported as ratio to β-actin and are the mean ± SEM of 4–7 animals/genotype.

### Statistical Analyses

Measurements were made by two or more investigators “masked” to the experimental groups. Statistically significant differences were analysed using SigmaPlot (v. 13; Systat Software, San Jose, CA, United States) or GraphPad Prism 7.0b (GraphPad Software; La Jolla, CA, United States) ^5^. Data sets were examined for normality (Shapiro–Wilk test) and equal variance (Brown–Forsythe test). Two-way ANOVA followed by Bonferroni’s or Holm–Sidak multiple comparisons test was used to assess a significant interaction between genotype and time or distance from the soma (Golgi staining). One-way ANOVA followed by Bonferroni’s multiple comparison test was used when three or more groups were compared. When only two groups were compared, significant differences were assessed by Student’s *t*-test. In the cases where the normality or equal variance assumptions were not met, a Kruskal–Wallis one-way ANOVA on ranks were used. Statistical significance was defined by *P* < 0.05 in all the analyses.

## Results

### Sleep Deficits in Pallid Mice

**FIGURE 1:**
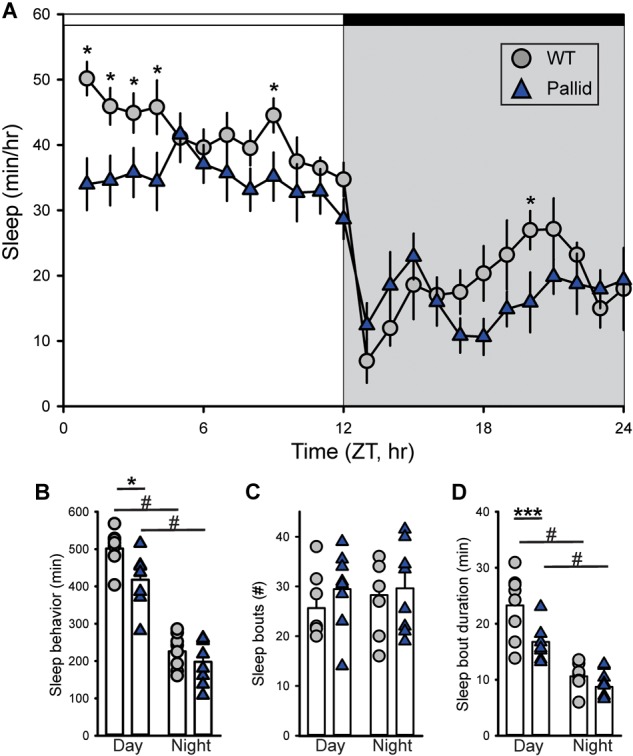
Altered timing of sleep behaviour in BLOC-1-deficient pallid mice. Video recording in combination with automated mouse tracking analysis software was used to measure immobility-defined sleep. **(A)** The temporal patterning of immobility-defined sleep was altered in the pallid mice, with their amount of sleep reduced in the early day. Running averages (1 h bin) of immobility-defined sleep in wild-type (WT) and pallid mice are plotted. ZT, Zeitgeber Time. 24-h profiles of sleep behaviour were analysed using a two-way ANOVA **(Table 1)** with genotype and time as factors, followed by Holm-Sidak’s multiple comparisons test, ^∗^*P* < 0.05. Data points are the mean ± SEM, *n* = 8 animals/genotype. **(B)** Pallid mice slept significantly less than WT during the day. **(C)** The number of sleep bouts was unaltered in mutant mice. **(D)** The duration of sleep in each bout was reduced for pallid mice in the day-time. Data are shown as the mean ± SEM and were analysed using the Student’s *t*-test to compare the results obtained between genotypes (^∗^*P* < 0.05, ^∗∗∗^*P* < 0.001) or within the genotype (^#^*P* < 0.05).

Behavioural phenotypes may be differentially affected by mutations in BLOC-1 subunits, hence, we first sought to determine how loss of BLOC-1 impacted the amount or temporal pattern of sleep behaviour in regular LD conditions. While both WT and pallid male mice exhibited robust daily rhythms in sleep behaviour, the mutants showed delayed sleep phase angle (about 4 h) and slept significantly less in the beginning of the sleep phase (Figure 1). Analysis of 1-h bins found overall a significant effect of time on sleep behaviour, with a significant interaction between the two factors (Table 1). The total amount of sleep (per 24 h) was significantly reduced in the mutants, mostly due to the decrease during the day, while at night this difference was minimal (Figures 1A,B and Table 1). Although the number of sleep bouts did not vary between genotypes (Figure 1C), their averaged duration was significantly shorter in pallid mice than in WT during the day (Figure 1D and Table 1). Thus, the pallid mice exhibited an abnormal sleep distribution, sleeping less during the day and displaying the most significant reduction at the beginning of the resting phase. These findings are suggestive of compromised sleep quality and deficits in the circadian timing system of the pallid mice.

**Table 1:** Lack of BLOC-1 negatively impacted sleep and circadian behaviour.

Two-way ANOVA			
**Genotype**	**Time (1 h bins)**		**Interaction**
*F*(1) = 1.888; *P* = 0.191	***F*(23) = 24.918; *P* < 0.001**		***F*(23) = 1.790; *P =* 0.015**

**Genotype**	**Time (12 h bins)**		**Interaction**
***F*(1) = 7.647; *P* = 0.010**	***F*(1) = 150.331; *P* < 0.001**		*F*(1) = 1.928; *P* = 0.176

**Sleep duration**	**WT**	**Pallid**	**Statistics**

24 h (min)	**727.5 ± 30.8**	**615.7 ± 41.7**	***t*(14) = 2.157; *P* = 0.049**
Day (min)	**501.7 ± 17.1**	**417.7 ± 25.1**	***t*(14) = 2.142; *P* = 0.046**
Night (min)	225.8 ± 16.4	197.9 ± 21.1	*t*(14) = 0.399; *P* = 0.694
Sleep bouts			
Day (#)	25.6 ± 2.3	29.4 ± 2.8	*t*(14) = 1.060; *P* = 0.306
Night (#)	28.2 ± 2.6	29.6 ± 3.1	*t*(14) = 0.337; *P* = 0.740
Day duration (min**)**	**24.5 ± 1.5**	**16.2 ± 0.8**	***t*(14) = 4.776; *P* = 0.0003**
Night duration (min)	10.6 ± 0.8	8.7 ± 0.9	*t*(14) = 1.584; *P* = 0.134

**Activity rhythms (LD)**	**WT**	**Pallid**	**Statistics**

Power	**60.1 ± 2.3**	**44.8 ± 4.5**	***t*(15) = 3.116; *P* = 0.007**
Activity (rev/h)	**404.8 ± 45.8**	**269.6 ± 40.5**	***t*(15) = 2.187; *P* = 0.045**
Alpha	415.7 ± 24.1	443.8 ± 47.5	*U*(15) = 29.5; *P* = 0.563
Precision (min)	–13.3 ± 4.5	–24.1 ± 3.6	*t*(15) = 1.911; *P* = 0.075
Fragmentation	**4.7 ± 0.3**	**6.5 ± 0.5**	***t*(15) = 3.356; *P* = 0.004**
% activity at night	**60.1 ± 2.3**	**44.8 ± 4.5**	***t*(15) = 2.350; *P* = 0.035**
Phase angle of entrainment	–4.4 ± 2.1	1.0 ± 8.5	*t*(15) = 0.521; *P* = 0.667
Activity rhythms (DD)	WT	Pallid	Statistics
Period	**23.7 ± 0.04**	**23.8 ± 0.05**	***t*(15) = 2.146; *P* = 0.047**
Power	**60.9 ± 3.2**	**40.0 ± 3.7**	***t*(15) = 4.170; *P* = 0.001**
Activity (rev/h)	373.4 ± 35.4	311.6 ± 38.7	*t*(15) = 1.187; *P* = 0.256
alpha	428.2 ± 29.9	390.2 ± 17.8	*t*(15) = 1.185; *P* = 0.256
Precision	**–20.2 ± 4.4**	**–45.7 ± 7.8**	***t*(15) = 2.821; *P* = 0.012**
Fragmentation	**5.7 ± 0.3**	**7.2 ± 0.5**	***t*(15) = 2.336; *P* = 0.033**
Phase angle	**–32.2 ± 4.2**	**32.0 ± 6.7**	***t*(15) = 0.983; *P* = 0.020**
Phase shift (CT16; min)	–100.3 ± 8.0	–132.3 ± 8.0	*U*(15) = 22.5; *P* = 0.122

*Two-way ANOVA followed by Holm—Sidak’s multiple comparisons test was used to evaluate the effects of genotype and time, and their interaction. Degrees of freedom are reported in parentheses. Data are reported as the mean ± SEM of 8–9 animals/genotype. Differences between genotypes were assessed by Student’s *t*-test or Mann-Whitney Rank Sum test. Bold values indicate statistically significant genotypic differences. Alpha = 0.05. WT, wild-type.*

### Disrupted Activity Rhythms in Pallid Mice

**FIGURE 2:**
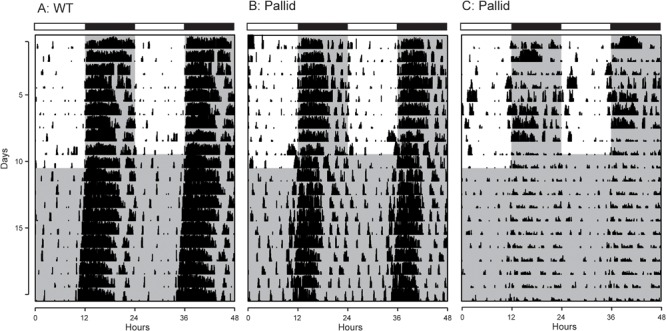
Examples of cage activity rhythms recorded from wild-type (WT) and pallid mice. Double-plotted actograms of cage activity in Light-Dark (LD) and Dark-Dark (DD) conditions. **(A)** Activity rhythm of age-matched WT control mouse. The pallid mice exhibited some heterogeneity in response with **(B)** showing the record from the least impacted and **(C)** showing the most impacted pallid mouse. The activity levels in the actograms were normalised to the same scale (85% of the maximum of the most active individual). All mice had a cage change on the day before the transition from LD to DD, to which some responded with a transient increase in activity. Each row represents two consecutive days, and the second day is repeated at the beginning of the next row. The white/black bar on the top of each panel indicates the 12:12 h Light-Dark cycle, and grey shading in the waveforms indicates the time of dark exposure.

**FIGURE 3:**
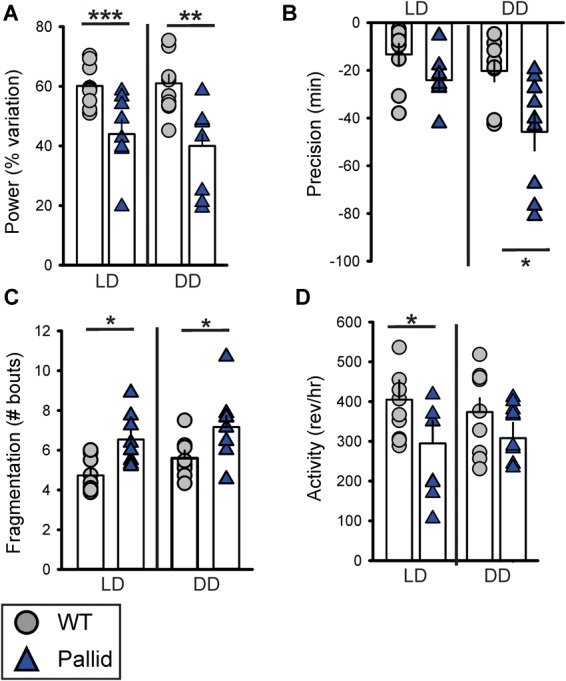
Locomotor activity rhythms are disrupted in BLOC-1-deficient pallid mice under both diurnal and circadian conditions. Quantification of the locomotor activity rhythms of wild-type (WT) and pallid mice under Light-Dark (LD) and Dark-Dark (DD) conditions. **(A–C)** A reduced strength of the activity rhythms was observed in pallid mice, as indicated by the significantly decreased power (% variation) as measured by the χ2 periodogram analysis **(A)**, lower precision (cycle-to-cycle variation in onset) **(B)** and highly fragmented activity **(C)**. The number of bouts of activity per 24 h cycle is reported as the amount of fragmentation of the daily activity cycle. **(D)** The total activity in LD was significantly lower in pallid mutants. Data are shown as the mean ± SEM and were analysed using the Student’s *t*-test to compare the results obtained for each genotype in LD or DD, ^∗^*P* < 0.05, ^∗∗^*P* < 0.01; ^∗∗∗^*P* < 0.001; *n* = 8–9 animals/genotype.

Next, the effects of BLOC-1 deficiency on diurnal and circadian rhythms (determined in LD and DD, respectively) of wheel-running behaviour were examined. The pallid mice displayed a high variability in both diurnal and circadian rhythms of activity ranging from mildly affected (6 mice; see for an example Figure 2B) to dramatically disrupted (3 mice; see for an example Figure 2C). Quantitative analyses of all the activity rhythms revealed a significantly reduced power (% Variation) of wheel running activity in both LD and DD (Figure 3A and Table 1). The precision (cycle-to-cycle variation in onset) was significantly altered in DD in the pallid mice (Figure 3B), which also showed higher fragmentation of the activity rhythms in comparison to WT (Figure 3C and Table 1). The mutants exhibited some reduction (27%) in voluntary motor activity (rev/h) in LD (Figure 3D and Table 1), with a higher percentage of activity in the daytime (Table 1) and reduced at night (25%). The period of the endogenous circadian cycle in the pallid mice was slightly but statistically significantly longer than in WT (Table 1). Finally, we measured the behavioural response to a brief light exposure at CT16, and found no significant difference in the magnitude of the light-induced phase delays between genotypes (Table 1). Thus, the pallid mice exhibit significant deficits in the generation of circadian rhythms in behaviour in physiological conditions.

### Behavioural Deficits in Pallid Mice

Behavioural deficits have been extensively characterised in the sandy mutant line (reviewed by Ghiani and Dell’Angelica, 2011; Glen et al., 2014; Bhardwaj et al., 2015b) but only to some extent in the pallid line (Spiegel et al., 2015). Thus, we evaluated the performance of pallid mutants in different behavioural tasks, beginning with the possibility of altered anxiety-like behaviour. Given the observed alterations in sleep and activity rhythms of the pallid mice (Figures 1–3), behavioural tests were carried out under specific points of the regular LD cycle.

**FIGURE 4:**
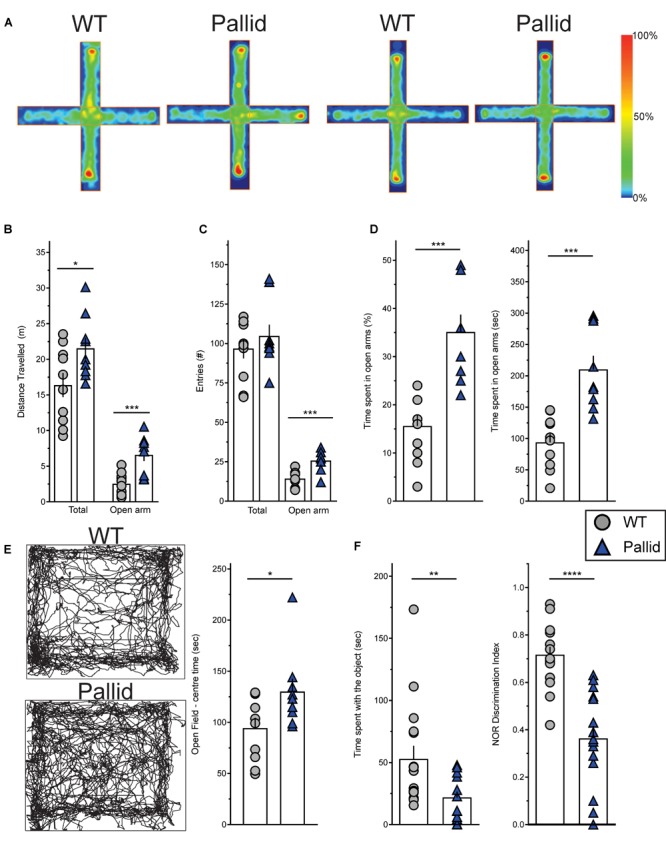
Altered behavioural performance in pallid mice. **(A)** Plus Maze Occupancy Plot Heat Maps: Representative heat maps (Horizontal = open arm; vertical = closed arm) of two distinct sets of wild-type (WT) + pallid showing the abnormal preference of the mutant mice for the open arm of the Elevated Plus Maze during the day/resting phase under white light (ZT2-10). The heat maps do not have absolute units, as the maximum signal is created relative to the “comparative maximum” of all mice compared in a given group. Heat maps comparisons can only be made within but not between the two groups. Heatmap scale is therefore relative, with blue representing the bottom 0–20% of time spent and red the top 80–100%. **(B)** The pallid mice travelled a greater distance than the WT, and most importantly, significantly more in the open arm. **(C,D)** Although the two genotypes had similar number of total entries, the pallid mice demonstrated a significant preference to enter **(C)** and stay **(D)** in the open arms. Student’s *t*-test ^∗^*P* < 0.05; ^∗∗∗^*P* < 0.001, *n* = 9–10 animals/genotype. **(E)** Open Field Occupancy Maps: Representative track plots (left panels) from individual WT and pallid mice showing that the mutant spent more time in the central part of the open field, further suggesting that they may have reduced levels of anxiety. Student’s *t*-test, ^∗^*P* < 0.05, *n* = 9–10 animals/genotype. **(F)** Pallid exhibited decreased novelty-induced behaviour, and spent less time exploring the objects (familiar and novel) compared to WT on day 3, and seemed almost unable to distinguish between novel and familiar objects, as suggested by the discrimination index (DI: Time spent with the novel object/Time spent with the familiar object + Time spent with the novel object) assessed on day 5. Student’s *t*-test, ^∗∗^*P* < 0.01 ^∗∗∗∗^*P* < 0.0001; *n* = 8–16 animals/genotype. Columns represent the mean ± SEM. Both the open field **(E)** and NOR **(F)** were administered during the animals’ active phase (ZT14–16) under dim red light.

Control (WT) and pallid mice were exposed to the EPM during the day/resting phase to assess anxiety-like behaviour. WT mice spent more time in the closed arms of the maze rather then in the open arms. In contrast, the pallid mice showed a significantly higher preference for the open arms and travelled a greater distance in this part of the maze compared to WT controls (Figures 4A,B and Table 2). Although the number of total entries did not differ between the two genotypes, the mutants had a higher number of entries in the open arm (Figure 4C) and also spent more time there both in relative (percentage of total time) and absolute terms (Figure 4D and Table 2). The impact of lack of functional BLOC-1 was further investigated using the OF test in the early night. The WT and pallid mice exhibited no differences in the distance or speed of movement, but, similarly to the behaviour observed in the EPM, the latter spent a significantly higher fraction of time (38%) in the centre of the OF arena (Figure 4E and Table 2). Overall, the pallid mice appear to have a reduced level of anxiety-like behaviour and no deficits in locomotor ability.

**Table 2:** Behavioural deficits in a mouse model of BLOC-1 deficiency.

EPM		WT (*n* = 10)	Pallid (*n* = 9)	Statistics
Total Distance (m)		16.4 ± 1.6	**21.5 ± 1.5**	***t*(17) = 2.292; *P* = 0.0349**
Total Entries (n)		96.8 ± 6.2	104.8 ± 7.1	*t*(17) = 0.844; *P* = 0.410
Open Arm				
Distance (m)		2.53 ± 0.5	**6.59 ± 0.9**	***t*(17) = 4.172; *P* = 0.0006**
Entries (n)		14.3 ± 1.6	**25.8 ± 2.1**	***t*(17) = 4.300; *P* = 0.0005**
Time	seconds	93.9 ± 13.4	**210.2 ± 21.7**	***t*(17) = 4.656; *P* = 0.0002**
	%	15.6 ± 2.3	**35.1 ± 3.6**	***t*(17) = 4.667; *P* = 0.0002**

**Open Field**		**WT (*n* = 10)**	**Pallid (*n* = 9)**	**Statistics**

Total Distance (m)		39.0 ± 2.0	46.4 ± 5.1	*U*(17) = 82; *P* = 0.140
Velocity (m/s)		0.07 ± 0.003	0.08 ± 0.008	*U*(17) = 80; *P* = 0.118
Centre Time (s)		94.3 ± 9.9	**130.1 ± 12.6**	***t*(17) = 2.244; *P* = 0.038**
Centre distance (m)		11.5 ± 1.6	12.8 ± 1.6	*t*(17) = 0.588; *P* = 0.564

**NOR**				

		**Time with the familiar object (s)**	**Time with the novel object (s)**	**Statistics**

WT (*n* = 16)		**13.6 ± 3.3**	**27.8 ± 7.5**	***U*(29) = 79.5; *P* = 0.034**
Pallid (*n* = 15)		18.7 ± 5.8	7.6 ± 1.8	*U*(29) = 91.5; *P* = 0.197

		**WT (*n* = 16)**	**Pallid (*n* = 15)**	

DI		0.72 ± 0.03	**0.36 ± 0.05**	***t*(29) = 5.846; *P* < 0.0001**

*Elevated plus maze test (EPM), open field test (OF) and novel object recognition (NOR) were administered to wild-type (WT) and pallid mice. Data are reported as mean ± SEM. Degrees of freedom are reported in parentheses. Differences between genotypes were assessed by Student’s *t*-test or Mann–Whitney Rank Sum test. Bold values indicate statistically significant genotypic differences. DI: discrimination index = time spent with the novel object/total time spent with both objects assessed; Alpha = 0.05.*

Next, the performance of the WT and pallid mice was examined in the NOR test during the night/active phase. In general, the mutants spent significantly less total time interacting with the objects than WT (Figure 4F). In the presence of a novel object, the WT mice spent significantly more time exploring this one over the familiar object but such preference was almost lost in the pallid mice (Table 2). Further analysis of their performance, by normalising the time spent with the novel object to the total amount of time spent with both objects (=discrimination index, DI), revealed a significantly lower DI for the pallid mice compared to the WT mice (Figure 4F and Table 2), suggesting that the mutants present memory and recognition capability deficits as they seem not to distinguish the novel from the familiar object.

Finally, both WT and mutants were challenged early in their active phase using the resident-intruder test, in which a stranger mouse is introduced into another mouse home cage. Two WT mice out of 6, but none of the seven pallid mice, exhibited some type of hostile comportment versus the intruder during the test period (5 min). Although no further analyses were performed, the apparent non-existence of aggressive behaviour might be a reflection of the lack of interest of the pallid mice for the surrounding environment, either objects or mice.

### Morphological and Cellular Abnormalities in the SCN of Pallid Mice

**FIGURE 5:**
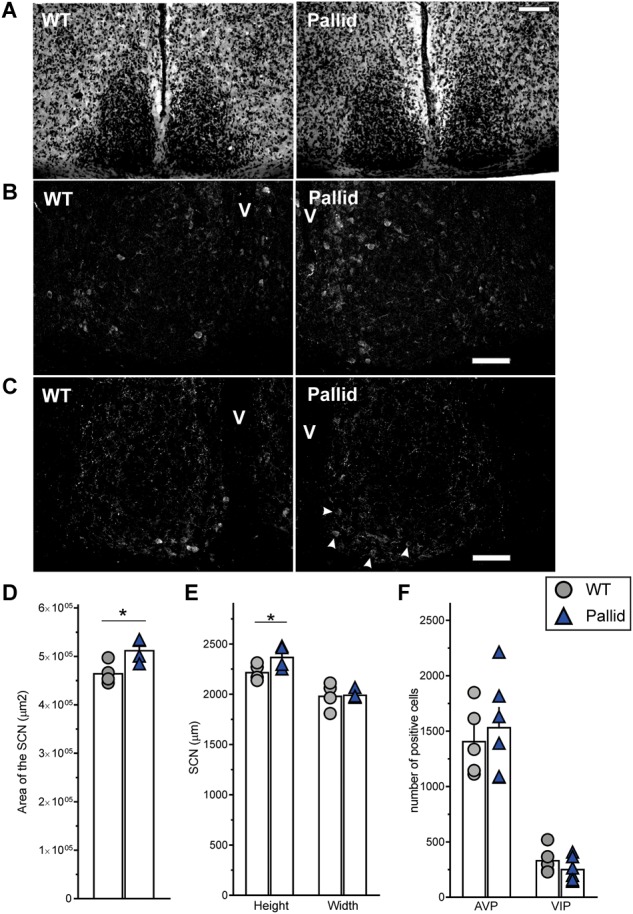
Subtle changes in the morphology of the suprachiasmatic nucleus (SCN) from pallid mice. Animals were perfused during the day (ZT6–8), and serial coronal slices containing the whole SCN were stained with cresyl violet (Nissl staining) **(A)** or with antibodies against the neuropeptides AVP **(B)** and VIP **(C**; arrowheads = VIP positive cell bodies in the pallid ventral SCN). **(D,E)** Gross morphometrical measurements of the Nissl-defined SCN revealed a significantly larger SCN area in pallid mice compared to wild-type (WT) mice **(D)**, mainly due to a significant change in the nucleus height but not in the width **(E)**. The area, height and width of both the left and right SCN were measured and averaged in 7 consecutive slices/animal containing the middle SCN. Individual data points represent the average of the left and right SCN values/animal (*n* = 4 animals/genotype). Student’s *t*-test, ^∗^*P* < 0.05. **(F)** No genotypic differences were found in the number of VIP and AVP positive neurones. Individual data points represent the number of positive cells counted per animal for each group (*n* = 5–6 animals/genotype). Columns represent the mean ± SEM. Scale bar = 100 μm.

To examine the possibility that loss of functional BLOC-1 altered the cellular organisation of the SCN, which might be causal to the observed sleep and circadian abnormalities, we examined the gross morphology of the SCN in Nissl-stained sections. The SCN from pallid mice consistently appeared larger than those from WT mice (Figure 5A). Further analysis of these sections revealed that the area of the SCN was about 10% greater in the mutants than in WT (Figure 5D), due to a slightly augmented height of the mutant SCN (Figure 5E). The neuropeptides VIP and AVP are thought to be an important part of the output of the SCN cell populations and are expressed in neuronal subpopulations within the SCN with a well-defined distribution. Stereological analysis found small non-significant changes, i.e., increased AVP-expressing neurones and a reduction (24%) in the number of VIP-expressing neurones in pallid mice (256.3 ± 45.1 cells) compared to WT (336.7 ± 43.8 cells, *P* = 0.231; Figures 5B,C,F). Some qualitative differences were observed in the organisation of the VIP-positive cell bodies in the SCN between WT and pallid. On the other hand, AVP seemed to have a stronger expression in the medial border of the pallid SCN (Figure 5B), however, quantification of the signal intensity in single cells revealed no differences between WT and mutant mice (WT: 675 ± 64; pallid: 650 ± 73, *P* = 0.207). Overall, these data suggest that the loss of BLOC1 elicits subtle morphological changes in the central circadian clock, which may trigger suboptimal outputs.

### Subtle Alteration in the Levels of PER2 in the BLOC-1-Deficient SCN During the Day

**FIGURE 6:**
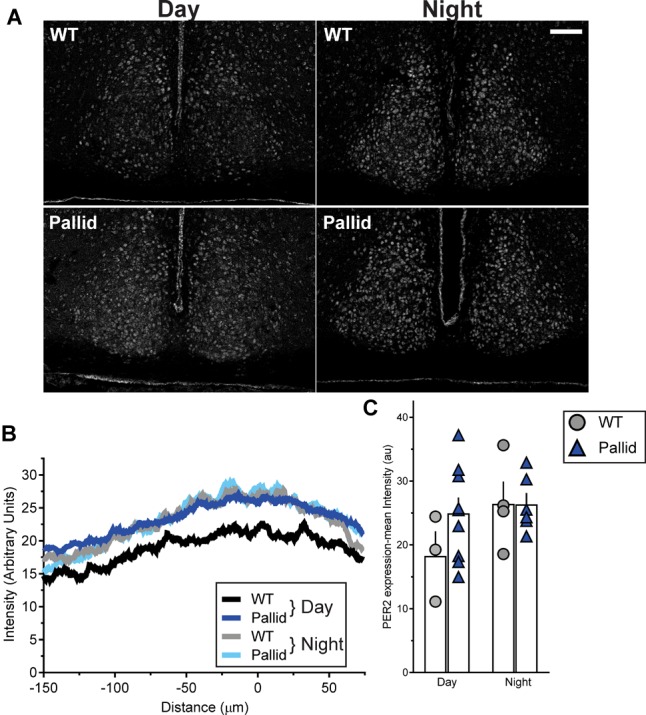
Altered expression of PER2 in the pallid suprachiasmatic nucleus (SCN) during the day. **(A)** Representative images of wild-type (WT) and pallid SCN stained with an antibody against PER2 displaying the relatively high expression of this clock protein during the day in pallid mice. **(B)** Densitometric analysis of the distribution of PER2 immunostaining intensity in the WT and pallid SCN revealed the characteristic higher expression of PER2 in WT animals at night (ZT14) compared to the day (ZT8). Such difference was lost in pallid mice. The intensity peaks of the profile plot of 2 consecutive sections containing the middle SCN/animal/time-point were aligned and then averaged to obtain a single curve per animal. Results are shown for the left SCN, but similar results were obtained for the right side. The graphed data are the average profile/genotype of 3–9 mice/genotype/time-point for the left SCN. **(C)** Measurements of the mean intensity of PER2 immunoreactivity in the SCN confirmed that PER2 expression in pallid mice during the day is comparable to WT and pallid levels at night. A single densitometric measurement was taken for each side/section. Individual data points represent the average of left and right value in 2 consecutive sections/animal containing the middle SCN. Columns represent the mean ± SEM of 3–9 animals/genotype/time-point. Two-way ANOVA followed by Bonferroni’s multiple comparisons test was used to evaluate the effects of genotype and time, and their interaction and revealed no significant differences [Genotype: *F*(1) = 1.193; *P* = 0.289; Time: *F*(1) = 2.492; *P* = 0.132; Interaction: *F*(1) = 1.23; *P* = 0.282]. Scale bar = 100 μm.

Circadian rhythms result from cell autonomous processes due to the precisely orchestrated interaction and activity of specific clock genes and their protein products (Takahashi, 2017). The levels of some of them (both at the mRNA and protein levels) vary with a circadian rhythm, including PER2, whose levels in the SCN peak during the late day/early night (around ZT12), and show a trough during the late night/early day (Wang et al., 2009). Thus, in light of the disrupted circadian timing system shown by the pallid mice (Figures 1–3), we examined PER2 immunoreactivity in the SCN in the day (ZT8) and night (ZT14). The WT showed the expected pattern of expression with PER2 levels increasing from ZT8 to ZT14, while the day/night difference was not so clear in the pallid SCN (Figure 6A). Analysis of the staining intensity and distribution confirmed that the levels of PER2 immunoreactivity during the day in the pallid SCN were not only higher than in WT, but comparable to those of both genotypes at night (Figures 6B,C). Similarly, the number of PER2 positive cells was increased in the pallid SCN (ZT8: 157 ± 11; ZT14: 208 ± 16) compared to WT [ZT8: 133 ± 25; ZT14: 177 ± 20; two-way ANOVA followed by Bonferroni’s multiple comparisons test, Genotype: *F*(1) = 2.471, *P* = 0.12; Time: *F*(1) = 7.756, *P* = 0.011; Interaction: *F*(1) = 0.00443, *P* = 0.83]. Thus, absence of BLOC-1 seems to interfer with the molecular clockwork at the single cell or network level in the SCN and its disruption could provide a possible mechanistic explanation for the disturbed sleep and activity rhythms observed in these mutants.

### Normal Neuronal Activity in the Central Clock in Pallid Mice

**FIGURE 7:**
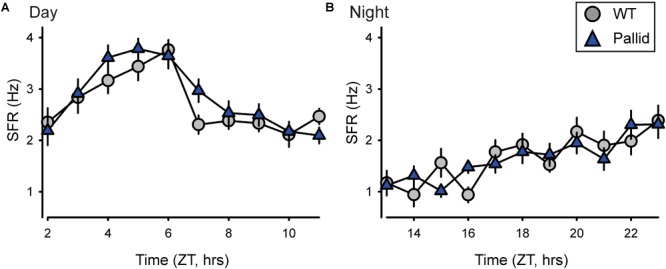
Loss of BLOC-1 does not impact the neuronal output of the suprachiasmatic nucleus (SCN) *in vitro*. **(A,B)** The rhythms in spontaneous firing rate (SFR) of SCN neurones were examined in wild-type (WT) and pallid mice using extracellular electrophysiological recordings in a brain slice preparation during the day and the night. ZT, Zeitgeber Time. The SFR were recorded and the results averaged to give a profile of **(A)** the day-time (ZT2–11; 29–33 neurones/h from 4 animals/genotype) and of **(B)** the night-time (ZT13–23; 20–24 neurones/h from 3 animals/genotype) firing rate. Data points are the mean ± SEM. Two-way ANOVA followed by Holm–Sidak’s multiple comparisons test was used to evaluate the effects of genotype and time, and their interaction revealed no significant differences during the day or the night (Supplementary Table 2).

The SCN in the anterior hypothalamus communicates with the rest of the body, in part, through a daily rhythm in electrical activity elicited by spontaneous neuronal activity. We examined the spontaneous firing rate (SFR) of the SCN neurones in WT and pallid mice and obtained a profile of firing rates during the day (ZT2–11) and night (ZT13–23) times. The timing of peak electrical activity of SCN neurones recorded during the day was not different between genotypes (Figure 7A and Supplementary Table 2). Similarly, there were no significant differences in the SFR peak or in the peak/trough ratio (Supplementary Table 2) between WT and mutants. In a separate set of recordings, no genotypic differences were found in the night-time SFR (Figure 7B and Supplementary Table 2). The SFR is a direct measure of the output of the SCN, these data indicate that the lack of BLOC-1 does not influence the normal rhythms in spontaneous neural activity and normal physiological functions of the central clock.

### Cytoarchitectural Abnormalities in the BLOC-1-Deficient Hippocampus

**FIGURE 8:**
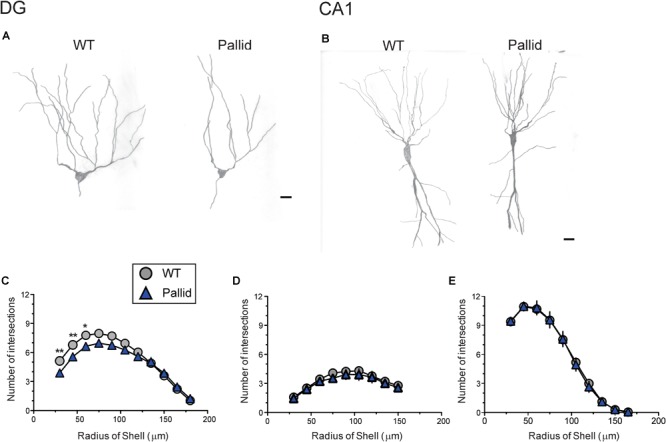
BLOC-1 deficiency results in defective dendritic arbourisation in the dentate gyrus (DG). **(A,B)** Representative camera lucida drawings of Golgi-impregnated granule cells in the DG (**A**) and pyramidal neurones in CA1 **(B)**. The morphology of granule cells in the pallid mice displayed a less elaborated arbour with fewer secondary and tertiary branches. WT, wild-type. **(C)** Sholl analysis performed on camera lucida drawings revealed a significant deficit in the number of processes crossing the concentric circles beginning at around 30 μm and continuing to about 75 μm from the soma in pallid granule cells as compared to WT, although decreased branch number could be observed up to 105 μm away from the soma. **(D,E)** No differences between genotypes were observed in the number of apical **(D)** or basal **(E)** dendrites of CA1 pyramidal neurones at any distance from the soma. Individual points are the mean ± SEM of *n* = 15 neurones/brain area/5–6 animals/genotype. Two-way ANOVA followed by Bonferroni’s Multiple Comparison test was carried out and showed a significant effect of genotype (Table 3), length of the neurites and their interaction. ^∗^*P* < 0.05, ^∗∗^*P* < 0.01. Scale bars = 25 μm.

The absence of BLOC-1 may impact regions receiving SCN outputs, including the hippocampus. Prior work had shown that primary cultures of hippocampal neurones from BLOC-1-deficient mice exhibit defective neurite outgrowth *in vitro* (Ghiani et al., 2010; Ma et al., 2011). Thus we examined the possibility of loss of BLOC-1 affecting the dendritic arbourisation of hippocampal neurones *in vivo* by analysing neuronal morphology in Golgi-impregnated WT and pallid brain slices. The cytoarchitecture of the granule cells in the mutants’ DG presented a relatively simpler morphology compared to those in WT mice (Figure 8A). Sholl analysis revealed that these cells in the pallid mice had on average about 15–24% fewer processes between 30 and 90 μm from the cell body compared to those in WT (Figure 8C and Table 3). The pallid granule cells showed a significantly lower number of branches in all the first three orders, with the most pronounced deficits being in the tertiary branches (Table 3). In general, these cells had fewer branching points, but only those related to the tertiary processes were statistically different between the two genotypes (Table 3). In contrast, the dendritic arbour of the pyramidal neurones in CA1 displayed a similar complexity in WT and pallid mice (Figure 8B), with no differences in the number of apical and basal dendrites or total branching points (Figures 8D,E and Table 3). The length of the processes in both cell types did not differ between WT and pallid mice (Table 3). These results not only validates previous findings from *in vitro* analyses, but, most importantly, raises the possibility that such defects may contribute to cognitive and behavioural deficits in the pallid mice.

**Table 3:** Morphological analyses of the dendritic arboures of hippocampal neurones uncovers deficits in P60 pallid Dentate Gyrus granule cells, suggesting that lack of BLOC-1 negatively affects neurite outgrowth also *in vivo*.

Two-way ANOVA				
	Genotype	Distance from the soma		Interaction
C	***F*(1) = 26.9; *P* < 0.0001**	***F*(10) = 145; *P* < 0.0001**		***F*(10) = 3.13; *P* = 0.0015**
D	*F*(1) = 2.08; *P* = 0.1535	*F*(9) = 14.0; *P* < 0.0001		*F*(9) = 0.08; *P* = 0.9998
E	*F*(1) = 0.02; *P* = 0.8998	*F*(10) = 240; *P* < 0.0001		*F*(10) = 0.06; *P* = 1.0000

**Number of dendrites**		**WT**	**Pallid**	**One-way ANOVA**

DG	Primary	**2.82 ± 0.14**	**2.12 ± 0.08^∗∗^**	***F*(5) = 97.14; *P* < 0.0001**
	Secondary	**4.97 ± 0.16**	**3.92 ± 0.08^∗∗∗∗^**	
	Tertiary	**5.96 ± 0.20**	**4.83 ± 0.16^∗∗∗∗^**	
CA1 Apical	Secondary	6.47 ± 0.64	5.91 ± 0.87	*F*(3) = 17.94; *P* < 0.0001
	Tertiary	2.04 ± 0.23	1.99 ± 0.30	
CA1 Basal	Primary	4.43 ± 0.12	4.25 ± 0.07	*F*(5) = 32.18; *P* < 0.0001
	Secondary	6.81 ± 0.26	7.07 ± 0.34	
	Tertiary	7.29 ± 0.38	7.33 ± 0.20	
Total branching points				Student’s *t*-test
DG		**7.99 ± 0.09**	**6.80 ± 0.39^∗∗^**	***t*(10) = 2.97; *P* = 0.0141**
CA1	Apical	7.59 ± 0.57	6.79 ± 0.92	*t*(8) = 0.74; *P* = 0.4829
	Basal	11.5 ± 0.29	10.9 ± 0.61	*t*(8) = 0.84; *P* = 0.4269
Length of dendrites				One-way ANOVA
DG	Primary	31.81 ± 1.47	32.64 ± 1.50	*F*(5) = 117.1; *P* < 0.0001
	Secondary	58.37 ± 2.63	64.80 ± 1.62	
	Tertiary	75.27 ± 1.34	76.24 ± 2.16	
CA1 Apical	Primary	109.8 ± 5.20	120.6 ± 10.5	*F*(5) = 27.56; *P* < 0.0001
	Secondary	63.82 ± 2.04	62.91 ± 2.02	
	Tertiary	50.25 ± 4.95	58.12 ± 4.68	
CA1 Basal	Primary	29.97 ± 2.85	31.14 ± 1.47	*F*(5) = 26.51; *P* < 0.0001
	Secondary	41.99 ± 0.75	48.91 ± 0.59	
	Tertiary	52.62 ± 4.00	56.25 ± 0.62	

*Two-way ANOVA followed by Bonferroni’s Multiple Comparison Test was used to evaluate the impact of genotype and distance on the number of processes of DG granule cells and CA1 pyramidal neurones crossing fixed-distance concentric circles (*n* = 5–6 animals/genotype), and revealed significant effects of both variables in the DG. A significant decrease in the number of dendrites and branching points was observed in pallid DG granule cells, with no differences in their length. Data are shown as the mean ± SEM of 12–15 neurones per animal/6 animals/region/genotype; One-way ANOVA followed by Bonferroni’s Multiple Comparison test: ^∗∗^*P* < 0.01, ^∗∗∗∗^*P* < 0.0001; differences in total branching points between genotypes were assessed by Student’s *t*-test. Degrees of freedom are reported in parentheses. Bold values indicate statistically significant genotypic differences. Alpha = 0.05, WT, wild-type.*

### Malfunctioning of Key Pathways in the Hippocampus of BLOC-1-Deficient Mice

**FIGURE 9:**
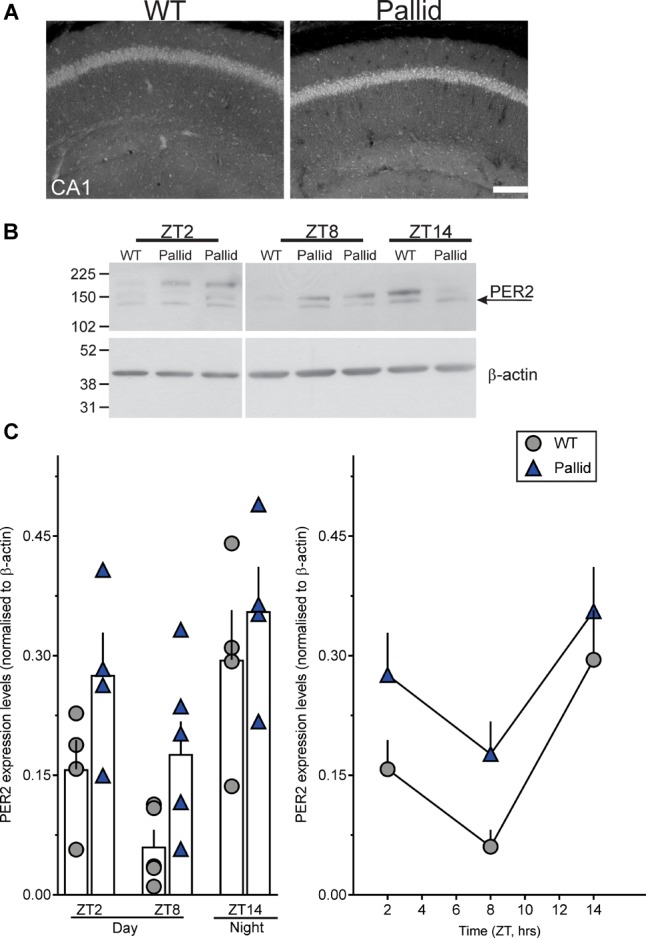
Altered PER2 expression levels in the pallid hippocampus during the day. **(A)** Representative images showing increased PER2 immunoreactivity in CA1 in pallid mice during the day at Zeitgeber Time (ZT) 8. WT, wild-type. **(B,C)** Daily rhythms in PER2 expression levels were observed in WT and pallid mice, with the latter exhibiting consistently higher expression levels of PER2 during the day (ZT2 and ZT8). **(B)** Representative immunoblots. **(C)** Elevated PER2 protein levels in whole hippocampal tissue lysates from pallid mice. Values derived from the densitometric analysis were corrected for the background, normalised to β-actin and represented as the means ± SEM of 4-7 animals/time-point/genotype. Two-way ANOVA followed by Bonferroni’s Multiple Comparisons test revealed statistically significant effects of both genotype and time (Table 4), but not of their interaction or significant genotypical differences at each time-point. Scale bar = 100 μm.

The hippocampus exhibits robust rhythms in the expression of the circadian clock protein PER2 as well as of the key signalling molecule CREB (Wang et al., 2009; Phan et al., 2011; Xia and Storm, 2017), and is highly sensitive to circadian disruption (Loh et al., 2015). Thus, we investigated whether lack of BLOC-1 affected the hippocampal expression levels of PER2 in the day (ZT2 & 8) and night (ZT14). The WT mice displayed the expected lower levels of PER2 during the late day compared to the night (Figure 9; Wang et al., 2009). Although a similar trend was evident in the mutants as well, upregulated levels of PER2 were observed in whole hippocampal tissue lysates from pallid mice compared to WT, more noticeably at ZT8 (Figures 9B,C). As judged from two-way ANOVA analysis, the PER2 protein level in hippocampus was significantly influenced not only by time (as previously reported; Wang et al., 2009) but also by genotype (Table 4).

**Table 4:** Lack of BLOC-1 alters the steady state protein levels of PER2 and the phosphorylated-to-total CREB ratio (pCREB/tCREB) in the adult hippocampus early in the day.

Two-way ANOVA			
	**Genotype**	**Time**	**Interaction**

PER2	***F*(1) = 6.968; *P* = 0.0153**	***F*(2) = 10.85; *P* = 0.0006**	*F*(2) = 0.242; *P* = 0.7868
pCREB/tCREB	***F*(1) = 4.868; *P* = 0.0381**	***F*(2) = 12.35; *P* = 0.0003**	*F*(2) = 0.977; *P* = 0.3922
pCREB	*F*(1) = 1.999; *P* = 0.1714	***F*(2) = 28.82; *P* < 0.0001**	*F*(2) = 0.532; *P* = 0.5949
tCREB	*F*(1) = 2.672; *P* = 0.1164	***F*(2) = 9.393; *P* = 0.0011**	*F*(2) = 0.051; *P* = 0.9501

**FIGURE 10:**
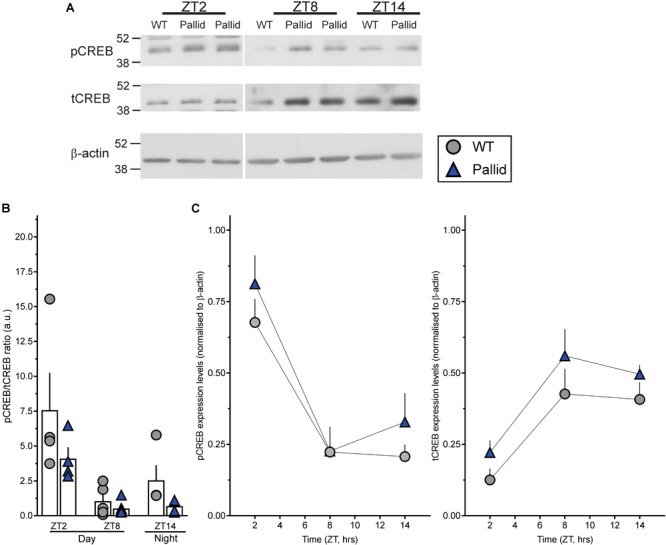
Impact of BLOC-1 deficiency on hippocampal CREB expression and phosphorylation during the day. **(A)** Representative immunoblots showing phosphorylated CREB (pCREB) and total CREB (tCREB) protein expression levels in wild-type (WT) and pallid mice during the day (ZT2 and ZT8) and night (ZT14). ZT, Zeitgeber Time. **(B)** The pCREB/tCREB ratio was decreased in pallid mice in the early day (ZT2), likely a consequence of the upregulated tCREB expression levels **(C)** in pallid mice compared to WT. Values derived from the densitometric analysis were corrected for the background and normalised to β-actin. These values were then used to calculate the pCREB/tCREB ratio/animal and are shown **(B)** as the mean ± SEM of 4–7 animals/genotype/time-point. **(C)** Data points are the mean ± SEM of 4–7 animals/genotype/time-point. Two-way ANOVA followed by Bonferroni’s Multiple Comparisons test was used to evaluate the effects of genotype and time on the observed changes. A significant effect of both variables on the pCREB/tCREB ratio was found (Table 4).

*Values derived from the densitometric analysis were corrected for the background, normalised to b-actin. These values were used to determine the pCREB/tCREB ratio in wild-type and pallid mice at each of the three time-points (Zeitgeber Times = ZT). Data are shown as the mean ± SEM of 4–7 animals/genotype/ZT in Figures 9, 10. Two-way ANOVA followed by Bonferroni’s Multiple Comparisons test was used to evaluate the effects of genotype and time on the changes observed in the expression levels of these proteins and revealed a significant effect of both variables on PER2 and the pCREB/tCREB ratio, but not of their interaction or significant genotypical differences at each time-point. Degrees of freedom are reported in parentheses. Bold values indicate statistically significant genotypic differences. Alpha = 0.05.*

The CREB signalling pathway exhibits robust rhythms and most importantly regulates PER2 expression levels (Koyanagi et al., 2011), hence, the levels of both pCREB and tCREB, as well as their ratio were determined at the same time-points (ZT2, 8, and 14) in WT and pallid mice. The ratio pCREB/tCREB was reduced in pallid mice as compared to WT at the three time-points (Figures 10A,B). Two-way ANOVA revealed a statistically significant effect of both genotype and time (Table 4). Interestingly, such reduction in pCREB/tCREB ratio was likely or at least in part a consequence of the elevated levels of tCREB observed in the pallid mice as compared to WT (Figures 10A,C, right panel). These observations suggest that lack of BLOC-1 elicits dysfunctional levels of both PER2 and tCREB in the hippocampus of the pallid mice during the resting phase, when important events such as memory consolidation are occurring.

## Discussion

In the attempt to better understand BLOC-1 involvement in brain functions, we sought to determine if a mouse deficient in this complex would exhibit disturbances in the sleep/wake cycle. Our findings revealed that lack of BLOC-1 greatly affected sleep quality and behaviour, with the strongest effects at the beginning of the resting phase. The mutant mice exhibited delayed sleep onset, less total sleep along with a more fragmented sleeping pattern, and weaker and more broken activity rhythms compared to WT mice. These altered behavioural phenotypes were accompanied by subtle structural changes in the SCN, the central circadian clock, and by elevated protein levels of the clock regulator PER2 during the day, but yet apparently normal neural activity rhythms were recorded. PER2 protein levels were also altered in the pallid hippocampus with, again, the most effect during the day, along with a reduced pCREB/tCREB ratio. In agreement with our *in vitro* findings, absence of BLOC-1 critically impacted the cytoarchitecture of the granule cells in the DG of the mutant hippocampus, which presented with a much simpler morphology compared to WT. Conversely, CA1 pyramidal neurones appeared normal. Altogether these data suggest that BLOC-1 absence negatively influence the circadian regulated outputs, such as the temporal patterns of activity and sleep, as well as cognitive performance.

BLOC-1 is a stable protein complex with a ubiquitous expression pattern (Falcón-Pérez et al., 2002; Moriyama and Bonifacino, 2002), including the ventral hypothalamus and the SCN (Lein et al., 2007), which has recently been linked to the molecular machinery for the coordination of the elongation of recycling endosomal tubules (Delevoye et al., 2016; reviewed by Dell’Angelica, 2016; Hartwig et al., 2018). Its functions in the central nervous system, however, remain incompletely understood. A plethora of studies has shown expression of BLOC-1 subunits in brain areas linked to cognitive functions (Talbot et al., 2004, 2006; Weickert et al., 2004; Feng et al., 2008; Tang et al., 2009; Larimore et al., 2013, 2014), and most importantly, drawn a complex interactome for this complex, with some binding partners having a strong association with IDD, including Disrupted in Schizophrenia (DISC)-1, MeCP2 and SNARE proteins (reviewed by Ghiani and Dell’Angelica, 2011; Hartwig et al., 2018). Decreased levels of the dysbindin subunit have been observed in the hippocampus and prefrontal cortex of post-mortem brain samples from schizophrenic individuals (Talbot et al., 2004, 2011; Weickert et al., 2008; Tang et al., 2009) as well as of the pallidin subunit transcript in human neurones carrying a mutation in *MECP2*, the gene that causes Rett Syndromes (Larimore et al., 2013). Genetic polymorphisms that generate loss of BLOC-1 function have been proposed to increase the risk of developing IDD (reviewed by Hartwig et al., 2018) perhaps in concert with other partner genes. These findings are in line with the hypothesis that neurodevelopmental disorders result from the interplay of more than one malfunctioning gene, explaining the overlapping phenotypes for some of these syndromes (Ghiani and Faundez, 2017 and references herein; Gandal et al., 2018). Recently, a 6-yr-old boy, who carries a mutation in dysbindin, was reported to display speech and other developmental delays (Bryan et al., 2017). This is the first case of an HPS-type 7 (mutation in *DTNBP1*, the gene that encodes dysbindin) affected individual with IDD and supports the contention that loss of function of BLOC-1 may negatively affect brain development and function(s).

In the present study, we report that BLOC-1 absence alters the temporal patterning of sleep behaviour in pallid mice. The mutants slept significantly less especially in the beginning of the resting phase and presented a more fragmented sleeping pattern (Figure 1). They also exhibited disrupted activity rhythms (Figures 2, 3), notwithstanding a high animal to animal variability in the impact of the mutation on the rhythms, ranging from to mildly irregular to severely impacted (Figure 2). The loss of rhythmicity was more pronounced when they were placed in constant darkness (DD) and the rhythms driven by the endogenous circadian timing system. We did not measure sleep with and without a running wheel, thus, it is possible that the sleep measurement would have been different in the presence of the running wheel (Thompson et al., 2016). Our observations on the pallid mutant mice are at variance with those reported for sandy mice, which exhibited normal activity rhythms in LD and DD, and a compromised circadian timing only when exposed to constant light (Bhardwaj et al., 2015b). Notably, highly abnormal activity/resting rhythms have been described in the *blind-drunk* mouse (Oliver et al., 2012), deficient in SNAP-25, a described binding partner of BLOC-1 (Ghiani et al., 2010). Similarly, other animal models of IDD, including fragile X (Zhang et al., 2008), Rett (Li et al., 2015), 2q23.1 deletion (Mullegama et al., 2015) and Angelman (Shi et al., 2015) syndromes, presented a disrupted circadian behaviour when placed in DD. Thus, a growing number of mouse models of IDD exhibit vulnerability to disruption of the circadian system. Importantly, these findings suggest that an altered circadian system can be a sensitive, although not a specific, indicator of a diseased nervous system.

The SCN in the anterior hypothalamus is the anatomical locus of the central oscillator in the circadian timing system. In the pallid mice, Nissl-staining indicated a small but significant change in the size of the SCN (Figure 4), yet the neural activity rhythms – the hallmark feature of SCN physiology – were normal (Figure 5). Thus, the observed structural alteration does not seem so critical as to impact this key centre’s function(s). Nonetheless, we observed some subtle alterations that could impair SCN function under certain conditions. For instance, persistent elevated levels of PER2 were observed during the day in the pallid SCN (Figure 6). Absence of BLOC-1 could somehow interfere with the rhythmic rise and fall in this clock gene levels in the SCN neurones, causing a delay in the resetting of the SCN molecular clock, as suggested by the small but significant lengthening of the free-running period of the circadian clock observed in the pallid mice (Table 2). In some ways, this phenotype resembles that of the *Afterhours* mice, which have reduced circadian strength and a longer period, likely due to sustained levels of the product of the clock gene *CRYPTOCHROME* (Godinho et al., 2007; Meng et al., 2008; Wegner et al., 2017). Given that we did see an impact of BLOC-1 loss on activity rhythms and clock gene expression, it was surprising to see normal neural activity rhythms. It is possible that they would have been disrupted in the intact organism, or that the deficits lie in SCN-driven outputs. Prior work in the *Fmr1* KO also failed to find significant changes in SCN firing rhythms, although the mutants exhibited behavioural disruption (Zhang et al., 2008). Conversely, in the *MeCP2* KO mouse, the SCN exhibited abnormal daily rhythm of the electrical activity that characterizes its neurones (Li et al., 2015) along with a smaller SCN and a decreased number of VIP-expressing neurones. Hence, the impact of the IDD-driving mutation on the SCN seems to vary with the gene/model and, specifically, in the pallid mice, the SCN circadian clock continues to be functional.

The hippocampus is one of the brain regions centrally involved in spatial memory and, more broadly, in the consolidation of short-term experiences into long-term memory. Furthermore, this area generates rhythms itself, which affect synaptic plasticity, learning and memory (Wang et al., 2009). At variance with the SCN, hippocampal expression of PER2 has been reported to peak in the early day and then decline reaching the lowest levels in the late day/early night (Wang et al., 2009). In the present study, we report that BLOC-1-deficient mice also exhibited a day/night difference in hippocampal PER2 protein levels but with relatively higher levels as compared to WT (Figure 9). These molecular rhythms likely underlie the well-known daily and circadian rhythms in recall of learned behaviour (see for example Holloway and Wansley, 1973; Chaudhury and Colwell, 2002; Eckel-Mahan et al., 2008; Gerstner and Yin, 2010). There is strong evidence that genetic disruption or environmental perturbations of these molecular oscillations have severe consequences on cognitive functions (see for example Karatsoreos et al., 2011; LeGates et al., 2012; Fernandez et al., 2014; Loh et al., 2015). For instance, a forebrain-specific disruption of the molecular clock (*Bmal*-/-) severely alters daily rhythms in learned behaviour without affecting the rhythms in wheel-running behaviour (Snider et al., 2016). The present data raise the possibility that the molecular clockwork in the hippocampus as well as rhythms in learning-involved pathways may be altered by lack of BLOC-1, although further in-depth investigation are needed. However, they do not allow us to determine whether the molecular clock is disrupted at the single cell level or there is a desynchronisation of the population rhythm; this distinction will have to await further studies.

Previously, we have proposed a role for BLOC-1 in neurite outgrowth in light of the deficits displayed by pallid hippocampal neurones *in vitro* (Ghiani et al., 2010). Here, we show that such deficit is present in adult pallid hippocampi and exclusively confined to the DG granule cells (Figure 9). Within the hippocampus, the strongest expression of pallidin and dysbindin has been observed in the inner molecular layer of the DG (Talbot et al., 2004, 2006; Larimore et al., 2013) and deficits in neuronal maturation in the DG have been observed in sandy mice (Nihonmatsu-Kikuchi et al., 2011; Wang et al., 2014). Mutations in a number of genes linked to IDD have now been shown to impact the development and cytoarchitecture of DG granule cells (e.g., Cope et al., 2016; Ito et al., 2017), some of which are part of the BLOC-1 interactome. For instance, neurite outgrowth is altered in PC12 cells transfected with a mutant form of DISC-1 (Ozeki et al., 2003) and in the DG of mice carrying a mutation in this gene (Kvajo et al., 2008, 2011). DISC-1, as the BLOC-1 subunits, is highly expressed in the DG, and has been proposed to functionally interact with BLOC-1 (Lee et al., 2015) as well as to play a role in cognition (Koike et al., 2006; Kvajo et al., 2008). The DG is a critical hub for memory formation and storage as its neurons process inputs from the enthorinal cortex, and send them to be stored in the CA3 region (Rolls, 2018). Disruption of sleep has been linked to the reduction in adult neurogenesis in the DG and suggested to underlie memory deficits (Mueller et al., 2015; Navarro-Sanchis et al., 2017). Thus, the deficits in granule cells’ dendritic arboures in the BLOC-1-deficient mice may provide a structural explanation for the impact of BLOC-1 on learning and memory.

In line with the above results, on a neurochemical level, we found strong evidence for altered pCREB/CREB ratio. It is well established that the transcription factor CREB controls gene expression essential for long-term synaptic plasticity and memory (e.g., Silva et al., 1998; Benito and Barco, 2010). In neurones, multiple signalling pathways stimulate gene expression through CREB phosphorylation at Ser133, and the resulting recruitment of CREB-binding protein/p300 coactivators, which activate transcription by acetylating nucleosomal histones. CREB phosphorylation has thus long been considered a critical event reflecting the activation of transcription by cAMP response elements. Although, there is also evidence that CREB can be activated independently of Ser133 phosphorylation (e.g., Impey et al., 1996; Briand et al., 2015). Prior work has shown that pCREB is rhythmic in the hippocampus (Eckel-Mahan et al., 2008) and the rhythms in this signalling pathway depend upon an intact SCN (Phan et al., 2011). In addition, we have reported that disruption of the circadian system with a mutation in *Per2* or by mistiming of feeding can disrupt pCREB and tCREB levels in hippocampal tissue (Wang et al., 2009; Loh et al., 2015). The impact of BLOC-1 absence was unusual in that we saw changes in the expression of tCREB, such that the pCREB/tCREB ratio was compromised in part due to increased tCREB rather than a decrease in pCREB. Finally, it is worth noting that a major function of this transcription factor is to enhance neuronal excitability (Lisman et al., 2018). The finding that tCREB levels are elevated in the pallid mice raises the possibility of altered electrical excitability in the mutant hippocampus and provides a strong biochemical explanation for the reduction in cognitive function in the BLOC-1-deficient mice.

Comorbidity between neurodevelopmental psychiatric disorder symptoms and sleep/wake cycle disruptions is well-established, and patients with the most disturbed sleep appear to have worse IDD-related symptoms (Schreck et al., 2004; Gabriels et al., 2005; Goldman et al., 2009). In particular, these individuals report delayed bedtime and frequent nocturnal awakenings (e.g., Devnani and Hegde, 2015; Elrod and Hood, 2015; Robinson-Shelton and Malow, 2016). These difficulties with the timing of sleep hint at a malfunctioning circadian timing system, hence melatonin, the circadian output hormone, is commonly used as a countermeasure to help sleep onset in these patients (Malow et al., 2012; Goldman et al., 2014; Rossignol and Frye, 2014; Schwichtenberg and Malow, 2015). Animal models are needed to better understand the mechanisms underlying the disrupted sleep/wake cycle to then develop new disease management strategies focused on restoring it. Although not without controversy, genetic variations that may compromise BLOC-1 function have been associated to an increased risk of developing neurodevelopmental psychiatric disorders, probably as one of the components of the triggering pathomechanisms (reviewed by Hartwig et al., 2018). Here we show that the BLOC-1-deficient pallid mice recapitulate several phenotypic and behavioural alterations observed in neurodevelopmental disorders. In this sense, this mutant may be well suited for pre-clinical studies looking to understand the underlying cellular and molecular mechanisms as well as to develop new treatment strategies.

## Author Contributions

DL, ED, CC, and CG conceived the hypothesis and experimental design of this study. FL, H-BW, DL, DW, OH, Y-SK, and CK performed the experiments. FL, H-BW, OH, DL, DW, Y-SK, AA, ED, CC, and CG analyzed the data. CC and CG wrote the paper with contribution of ED and the other authors.

## Conflict of Interest Statement

The authors declare that the research was conducted in the absence of any commercial or financial relationships that could be construed as a potential conflict of interest.

## Footnotes

1 https://www.jax.org/strain/000024

2 https://www.jax.org/strain/000664

3 http://www.el-temps.com/principal.html

4 http://rsb.info.nih.gov/ij/

5 www.graphpad.com
