# Supplementary material for: Sleep/Wake Disruption in a Mouse Model of BLOC-1 Deficiency
Source: Front Neurosci. 2018 Nov 15;12:759. doi: 10.3389/fnins.2018.00759 (PMC6249416; doi:10.3389/fnins.2018.00759)
Supplement: Supplementary file 1 [file Table_1.DOCX]

**Supplementary Table 1.** Antibodies used in the present study. WB: western blot; IHC: immunohistochemistry; AVP: arginine-vasopressin peptide; PER2: period2; pCREB: phosphorylated cAMP-responsive element binding; tCREB: total CREB; VIP: vasoactive intestinal peptide.

| Target | Clone | Specie | Dilutions | | Cat. Number | Company |
| --- | --- | --- | --- | --- | --- | --- |
|  |  |  | WB | IHC |  |  |
| AVP |  | Guinea Pig |  | 1:500 | T-5048 | Peninsula Lab Int Inc. |
| PER2 |  | Rabbit | 1:1000 | 1:500 | PER21-A | Alpha Diagnostic Int |
| pCREB |  | Rabbit | 1:1,000 |  | 06-519 | EMD-Millipore |
| tCREB |  | Rabbit | 1:500 |  | 06-863 | EMD-Millipore |
| VIP |  | Rabbit |  | 1:1000 | 20077 | ImmunoStar |
| β-actin | AC-15 | Mouse | 1:10,000 |  | A5441 | Sigma-Aldrich |

**Supplementary Table 2. Spontaneous neuronal activity in the SCN is not affected by loss of BLOC-1.** Spontaneous firing rate (SFR) of the SCN neurones was measured during the day (ZT 2-11) in WT and pallid (n=29-33 neurones/hr from 4 animals/genotype) and during the night (ZT 13-23; n= 20-24 neurones/hr from 3 animals/ genotype). Values from cells/hr were averaged to obtain the day and night profiles and are reported as the mean ± SEM. Two-way ANOVA followed by Holm-Sidak's multiple comparisons test was used to evaluate the effects of genotype and time, and their interaction. Degrees of freedom are reported in parentheses. Differences between genotypes were assessed by Student’s *t*-test or Mann-Whitney Rank Sum test. Alpha = 0.05.

| Two-way ANOVA |  |  |
| --- | --- | --- |
| Genotype | Time (Day) | Interaction |
| *F*(1)=1.334; *P*=0.249 | *F*(9)=14.318; *P*<0.001 | *F*(9)=1.591; *P*=0.338 |
|  |  |  |
| Genotype | Time (Night) | Interaction |
| *F*(1)=1.156; *P*=0.937 | *F*(1)=8.228; *P*<0.001 | *F*(1)=1.156; *P*=0.319 |

|  | |  |  |
| --- | --- | --- | --- |
| Day | WT | Pallid | Statistics |
| Peak of electrical activity (ZT, hr) | 5.5 ± 0.3 | 5.7 ± 0.2 | *t(6)*=0.655; *P*=0.537 |
| SFR Peak (Hz) | 3.8 ± 0.1 | 3.7 ± 0.1 | *t(6)*=0.773; *P*=0.469 |
| Peak/Trough ratio (Hz) | 1.9 ± 0.1 | 2.4 ± 0.3 | *U(6)*=2.0; *P*=0.114 |
|  |  |  |  |
| Night |  |  |  |
| Average SFR (Hz) | 1.6 ± 0.2 | 1.7 ± 0.2 | *t(20)*=0.044; *P*=0.965 |
